# Supplementary material for: A Critical View of the Application of the APEX Software (Aqueous Photochemistry of Environmentally-Occurring Xenobiotics) to Predict Photoreaction Kinetics in Surface Freshwaters
Source: Molecules. 2019 Dec 18;25(1):9. doi: 10.3390/molecules25010009 (PMC7017383; doi:10.3390/molecules25010009)
Supplement: Supplementary file 1 [file molecules-25-00009-s001.zip › 3.2.4_gcc-4.4.0.zip!/3.2.4_gcc-4.4.0/3.2.4_gcc-4.4.0/doc/octave/html/interpreter/Cell-Array-Objects.html]

Cell Array Objects - Untitled


Previous: Data Structure Objects,
Up: Built-in Data Types


---

#### 3.1.5 Cell Array Objects

A Cell Array in Octave is general array that can hold any number of
different data types.

See Cell Arrays, for more information.
